# Employing Raman Spectroscopy and Machine Learning for the Identification of Breast Cancer

**DOI:** 10.1186/s12575-024-00255-0

**Published:** 2024-09-12

**Authors:** Ya Zhang, Zheng Li, Zhongqiang Li, Huaizhi Wang, Dinkar Regmi, Jian Zhang, Jiming Feng, Shaomian Yao, Jian Xu

**Affiliations:** 1 Division of Electrical and Computer Engineering, College of Engineering, Louisiana State University, Baton Rouge, LA 70803 USA; 2 Division of Computer Science & Engineering, College of Engineering, Louisiana State University, Baton Rouge, LA 70803 USA; 3 Department of Comparative Biomedical Science, School of Veterinary Medicine, Louisiana State University, Baton Rouge, LA 70803 USA

**Keywords:** Raman spectroscopy, Machine learning, Late-stage breast cancer; Cancerous and non-cancerous tissue classification

## Abstract

**Background:**

Breast cancer poses a significant health risk to women worldwide, with approximately 30% being diagnosed annually in the United States. The identification of cancerous mammary tissues from non-cancerous ones during surgery is crucial for the complete removal of tumors.

**Results:**

Our study innovatively utilized machine learning techniques (Random Forest (RF), Support Vector Machine (SVM), and Convolutional Neural Network (CNN)) alongside Raman spectroscopy to streamline and hasten the differentiation of normal and late-stage cancerous mammary tissues in mice. The classification accuracy rates achieved by these models were 94.47% for RF, 96.76% for SVM, and 97.58% for CNN, respectively. To our best knowledge, this study was the first effort in comparing the effectiveness of these three machine-learning techniques in classifying breast cancer tissues based on their Raman spectra. Moreover, we innovatively identified specific spectral peaks that contribute to the molecular characteristics of the murine cancerous and non-cancerous tissues.

**Conclusions:**

Consequently, our integrated approach of machine learning and Raman spectroscopy presents a non-invasive, swift diagnostic tool for breast cancer, offering promising applications in intraoperative settings.

## Background

Breast cancer is one of the most prevalent cancers diagnosed in females in the United States; new breast cancer accounts for 31% of estimated various cancers, and mortality of all types of breast cancer is the second out of all female cancerous diseases [1]. Regular breast cancer screening, including clinical breast exams and mammograms, has enhanced early detection rates and is crucial for the prognosis and treatment planning of surgery, radiation therapy, chemotherapy, and the latest targeted therapies [2]. The diagnosis of breast cancer is not only via imaging but also associated with histopathological analysis of the patient’s tissue, which is invasive and painful [3]. Haka and colleagues compare the pathological reports to the breast cancer Raman spectra classification, achieving over 90% sensitivity and specificity [4]. For late-stage breast cancer and non-surgical breast cancer cases, accurately assessing prognosis becomes particularly critical in guiding therapeutic decisions, personalizing treatment plans, and improving patient outcomes [5].

The most common imaging modalities for breast cancer detection are mammograms, magnetic resonance imaging (MRI), ultrasound, and fluorescence imaging [6–11]. However, those approaches have various limitations, which impact their diagnostic efficacy. In particular, mammography exhibits reduced sensitivity in the presence of dense mammary tissue, a condition that can mask potential malignancies. MRI, while offering detailed tissue visualization, incurs excessive costs and often necessitates contrast agents, posing potential risks and discomfort for patients. Ultrasound, despite its non-invasive nature, is heavily dependent on the operator’s skill and experience, leading to variability. Indocyanine Green (ICG)-)-assisted near-infrared imaging illustrated the ability to identify cancerous and non-cancerous tissues. Researchers applied ICG to the sentinel lymph node for early breast cancer detection, achieving solid diagnostic results [12–14]. However, fluorescence imaging is exogenous imaging in nature, which brings contamination to the tissues and the fluorescent dye may cause side effects to the patients [15–18]. The Raman imaging system can not only avoid this problem by directly detecting the tissues without any processes but also provide chemical component information [19]. Moreover, compared to conventional imaging methods, optical imaging and spectroscopy methods like Raman spectroscopy are minimally invasive and offer the advantage of quicker, more specific, and more sensitive cancer detection [20]. Raman spectroscopy serves as a molecular fingerprinting technique, evaluating vibrational and rotational energies to examine intermolecular functional groups and their molecular structures. This method facilitates rapid molecular analysis of tissues in vivo and ex vivo, suitable for biopsy evaluations or laboratory investigations, owning to its non-destructive approach [19]. It can discern variations in molecular compositions and structures between normal and breast cancer tissues, making it a powerful tool for identifying cancerous changes with precision [4, 21–25]. Traditional approaches to analyzing Raman Spectra mainly involve manual feature selection and linear statistical models, which may not capture the high-dimensional, nonlinear relationships inherent in the data. These limitations can hinder the accuracy and robustness of cancer diagnostics based on Raman spectroscopy, making it difficult to differentiate between various types and stages of cancer effectively.

Machine learning is currently being explored in diverse cancer diagnosis and classification fields. By feeding large amounts of biomedical data (e.g., cancerous Raman spectra), machine learning algorithms can autonomously deliver diagnostic outcomes and rapidly and effectively explore hidden valuable features related to cancerous tissues [26]. Machine learning applications have been used for breast cancer analysis, achieving effective results in previous research [27]. Kneipp et al. utilized PCA and K-means algorithms to differentiate between secretions from normal and cancerous breast duct epithelial cells [28]. Wu et al. achieved over 90% accuracy in classifying luminal and basal-like breast cancer subtypes using SVM-based algorithms that analyzed pathway-based biomarkers linked to specific genes [29]. There are few studies about late-stage breast cancer diagnosis using Raman spectroscopy, especially in the mouse model. Kast et al. applied principal component analysis - discriminant function analysis (PCA-DFA) for breast cancerous tissue and normal tissue classification [30]. Though some human breast cancer tissues were studied with the Convolutional Neural Networks (CNN) model [31, 32], there were few animal breast cancer studies reported. Animal models may also provide useful insights for clinical diagnoses; chemical components and contents could provide a consistent comparison to the human model. To our knowledge, this is the first study of CNN-enhanced signal processing for Raman spectroscopy-assisted animal breast cancer diagnosis for classification and feature extraction.

Evaluating the efficacy of machine learning-assisted Raman spectroscopy in diagnosing late-stage breast cancer in mouse models is imperative [33]. Raman spectroscopy can be performed to provide detailed molecular-level information about the tissues’ chemical composition, enabling precise differentiation between cancerous and non-cancerous mammary tissues [21]. To the best of our knowledge, this study marks a pioneering effort in employing Raman spectroscopy, enhanced with machine learning algorithms—specifically, Random Forest, Support Vector Machine, and Convolutional Neural Networks—for the detailed analysis of the stage IV breast cancer tissues.

## Materials and Methods

### Cell Lines and Medium

4T1 (ATCC ^®^, CRL-2539^™^), a mouse breast cancer cell line that mimics human Stage IV breast cancer, was applied in this study to perform the nonsurgical model of breast cancer. The 4T1 cells were cultured in RPMI-1640 Medium (ATCC ^®^, 30-2001^™^) with 1% Penicillin-Streptomycin (Fisher Scientific, Gibco™ 15-140-122), 10% Fetal Bovine Serum (Fisher Scientific, Gibco™ A5256801) in T75 flasks in the sterile and humidity incubator, setting 37 °C, 5% Carbon Dioxide.

### Animal, Raman Spectroscopy System, and Raman Measurement

#### Animal Model

To generate the allograft animal model, the 4T1 cells were subcutaneously injected into 20 six- to eight-week-old athymic nude Nu/J female mice (IMSR_JAX:002019). Each mouse was injected with 2 × 10^6^ 4T1 cells resuspended in 100 µL phosphate-buffered saline (PBS). When the tumor volume was about 50 mm^3^, the mice were euthanized via isoflurane as the first form and cervical dislocation as the second form. This study was approved by the Institutional Animal Care and Use Committee of Louisiana State University (The protocol number: IACUC#23–061), and all operations followed the guidelines on animal research.

#### Raman Spectroscopy System and Raman Measurement

**Fig. 1:**
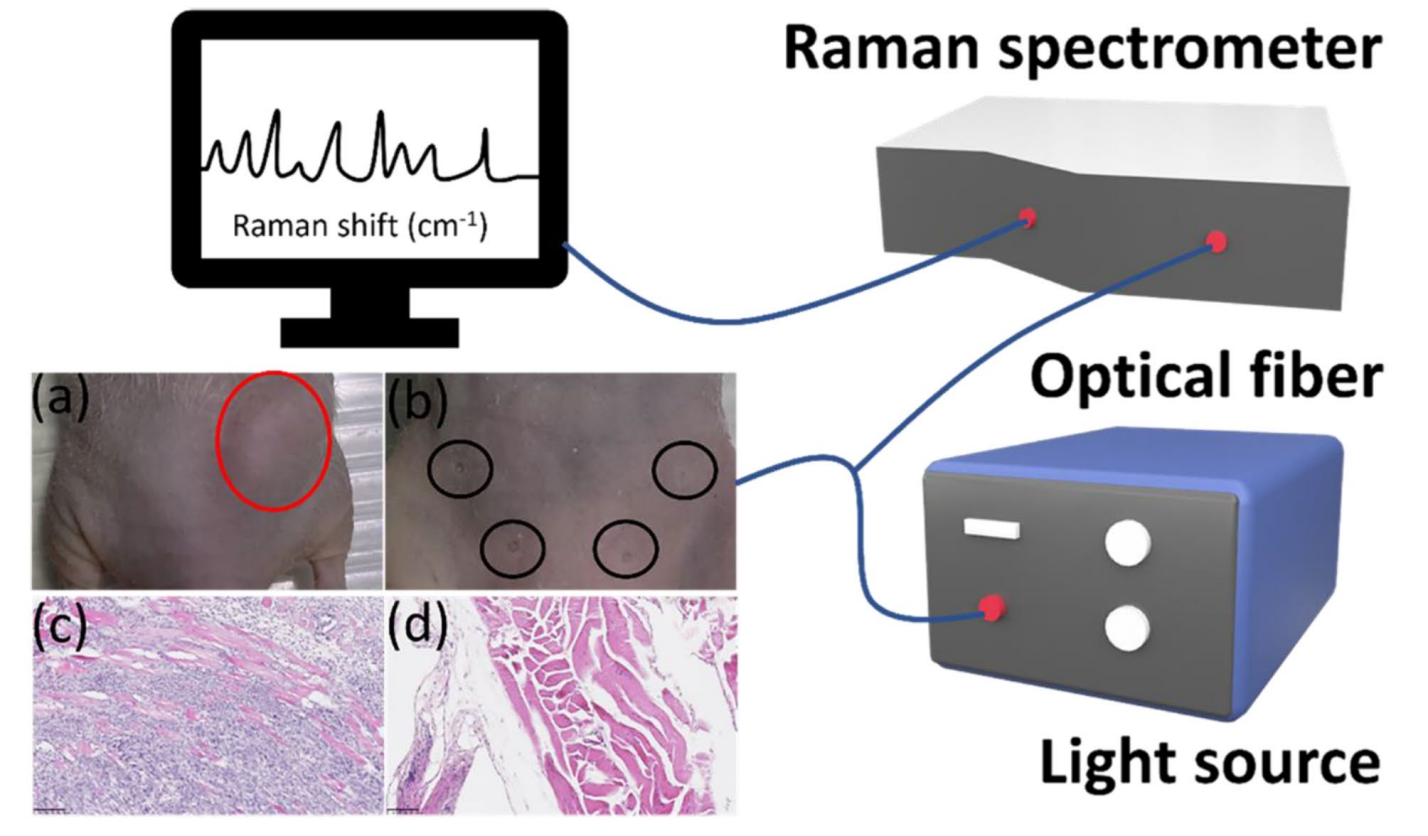
Schematic diagram of Raman system in a murine cancer model. (**a**) tumor; (**b**) normal breast; (**c**) tumor with H&E staining; (**d**) normal breast with H&E staining

The Raman Spectroscopy system used in this study consists of Raman Endoscopic Probe (EmVision LLC. Loxahatchee, Florida, United States), QE Pro spectrometer (Ocean Optics, Inc. Orlando, Florida, United States), and 785 nm laser diode source (Turnkey Raman Laser-785 Series, Ocean Optics Inc., Orlando, Florida, United States) connected with a desktop computer to perform Raman data acquisition via OceanView Software with 3 s exposure time [34, 35]. Once the mice were euthanized, the tumor was resected for collecting Raman spectra (Fig. 1). Eighteen female mice were used for data acquisition, 959 Raman spectra were collected from the tumor, and 1075 Raman spectra were collected from the breast (Fig. 1a and b). Breast cancer specimens and normal mammary tissues were examined histologically after hematoxylin and eosin (H&E) staining (Fig. 1c and d).

### Data Processing

Preprocessing of the collected raw Raman data is of chief importance as the data contain multiple noises [36–38]. The preprocessing was guided by the following steps: autofluorescence backgrounds of raw data were removed by asymmetric least squares fitting [39, 40]; Savitzky-Golay smoothing filter was applied to remove the noise without changing the main peak intensity [41]; normalizing the Raman data from 0 to 1 via mapping the minmax function. The procedures were implemented in MATLAB (version R2022a, MathWorks Inc., Natick, MA, USA).

### Data Analysis via Random Forest (RF) Model, Support Vector Machine (SVM) Model, and Convolutional Neural Network (CNN)

**Fig. 2:**
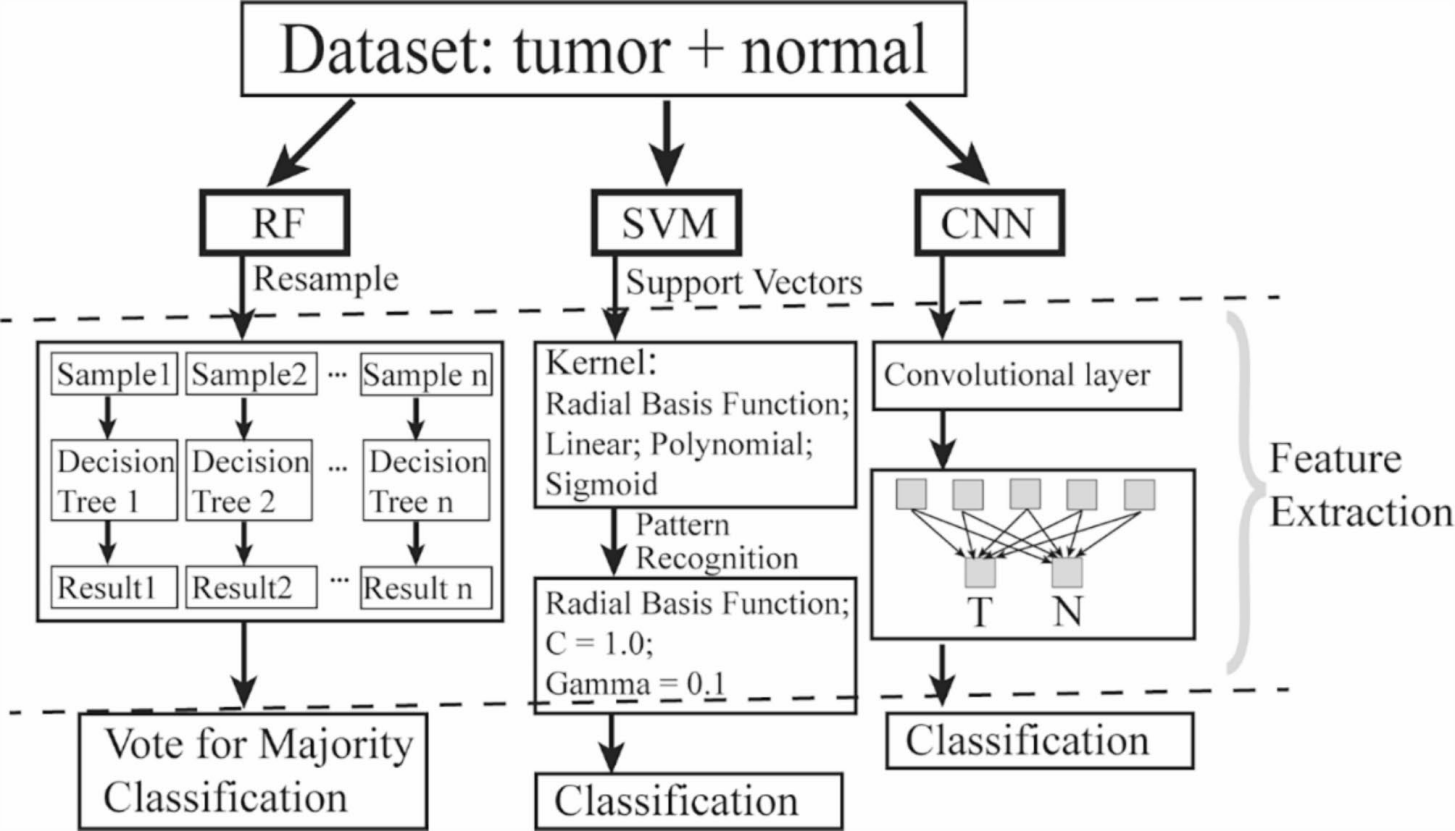
Structures of RF, SVM, and CNN models

Figure 2 demonstrated the structures of RF, SVM, and proposed CNN models [35, 42, 43]. Random Forest is based on decision trees, and each decision would achieve a result after resampling. The majority vote finalizes the classification performance. SVM uses support vectors/margins and kernel of radial basis function/ linear/ polynomial/ sigmoid for the classification. The CNN model has one convolutional layer and one fully connected layer. The CNN model utilized a kernel size of five coupled with a stride of two. It employed the binary cross-entropy loss function (specifically BCEWithLogitsLoss) and was configured with a learning rate of 0.01, a momentum setting of 0.9, and a weight decay parameter set to 0.00004. For optimization, the model leveraged Stochastic Gradient Descent (SGD).

## Results

### H&E Staining

Figure 1c reveals invasive cancerous cells penetrating the normal muscle tissue, accompanied by an increased density of blood vessels. In contrast, Fig. 1d depicts normal mammary tissue characterized by regular arrangements of muscle and fat tissue, illustrating the typical structure and composition of healthy mammary tissue. This heightened vascular presence suggests a greater consumption of nutrients by the cancerous tissue compared to normal tissue, indicative of the aggressive nature of invasive breast cancer. The observed pathological features in these images have been validated by experienced pathologists, confirming the diagnostic significance of these findings.

### Raman Spectra

**Fig. 3:**
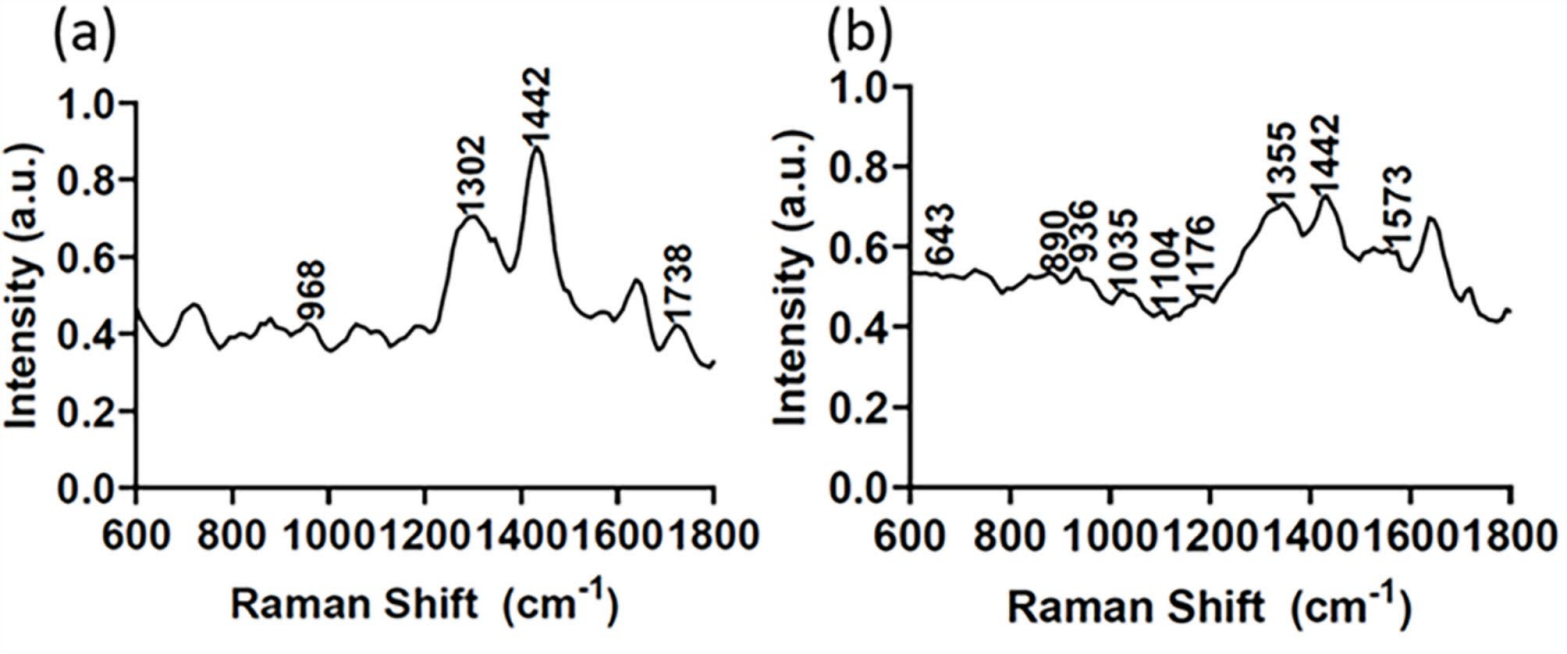
Averaged Raman spectra of normal tissues (**a**) and breast cancer (**b**) with their respective remarkable peaks

Figure 3 shows the normalized average Raman spectra of healthy and cancerous mammary tissues in the range of 600–1800 cm^-1^. The pronounced lipid content (e.g., 968, 1442, and 1738 cm^-1^) exists in the healthy mammary tissue (Fig. 3a); conversely, breast cancer tissue’s elevated intensity of proteins (e.g., 890 cm^-1^ and 1104 cm^-1^) and decreased 1442 cm^-1^ band owing to the contribution of lipids [44] (Fig. 3b). The increased protein content and altered lipid profiles in cancerous tissues indicate the metabolic reprogramming associated with cancer progression [3, 30].

### Classification Performances of Machine Learning Models

**Fig. 4:**
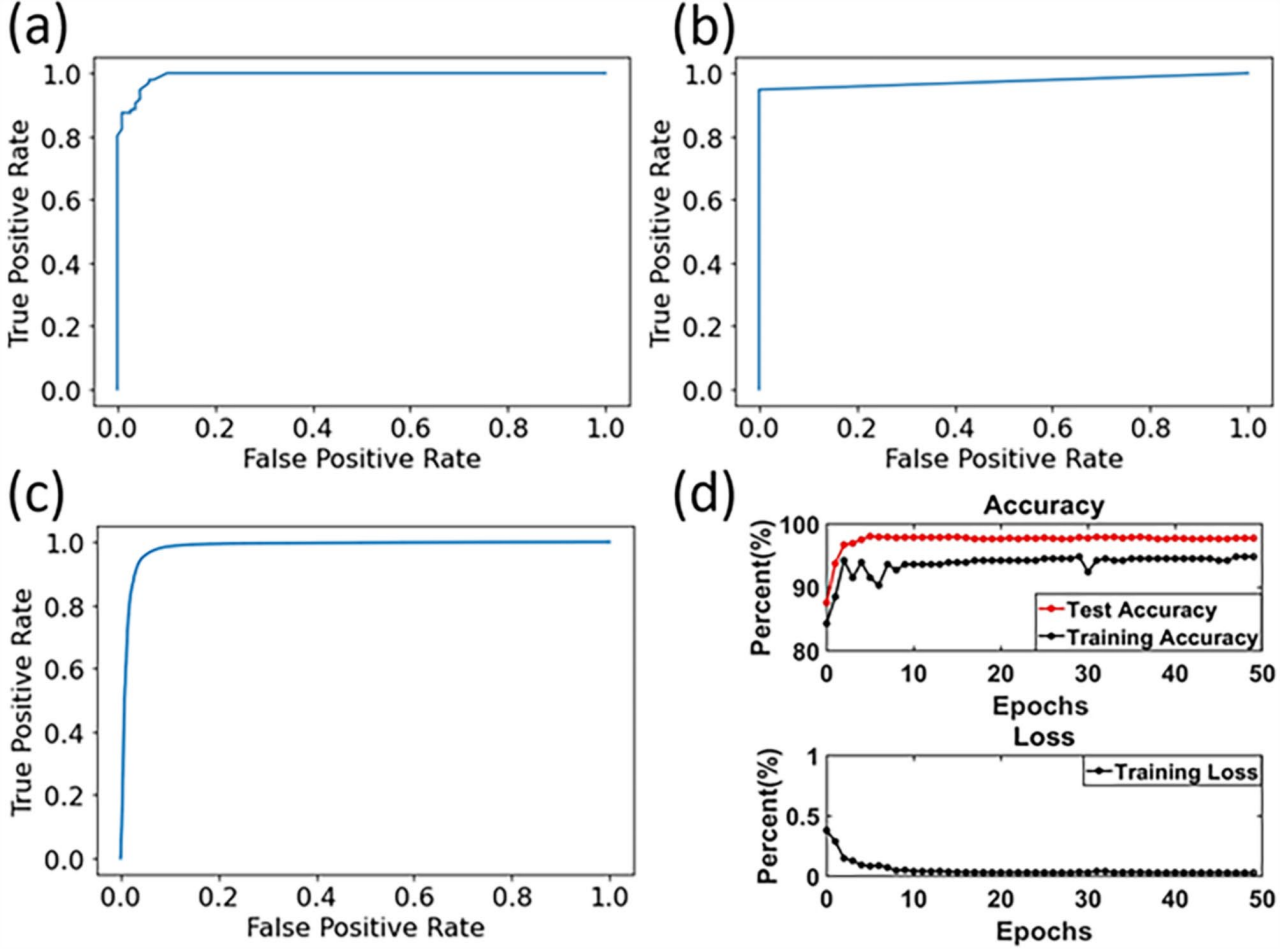
ROC curves of RF (**a**), RBF-SVM (**b**), and CNN (**c**); (**d**) accuracy/loss to epochs curve of CNN model

For RF classification, our study allocated 80% of the data to train the model and reserved the remaining 20% for testing its efficacy. The model demonstrated an average accuracy rate of 94.47%. It achieved a specificity of 96.73% and a sensitivity of 92.4%. The receiver operating characteristic (ROC) curve of RF is shown in Fig. 4a. The area under the curve (AOC) was 0.9849.

In the exploration of SVM classification utilizing the Radial Basis Function (RBF) kernel, our study partitioned the data, allocating 80% for training and the remaining 20% for testing. The RBF-SVM model demonstrated a commendable average accuracy of 96.76%, with an impressive specificity of 98.74% and a sensitivity of 94.90%. The receiver operating characteristic (ROC) curve of RBF-SVM is shown in Fig. 4b. The area under the curve (AOC) was 0.9722. We also test other kernels of SVM models. The model’s performances are shown in Table 1.

For CNN classification, our study applied 80% of the data for training with 50 epochs and the rest for testing. The CNN model achieved an average accuracy of 97.58%, with unparalleled specificity and sensitivity of 99.51% and 95.65%, respectively. The receiver operating characteristic (ROC) curve of CNN is shown in Fig. 4c. The area under the curve (AOC) was 0.9842. The accuracy became convergent after ~ 35 epochs (Fig. 4d).

**Table 1:** Classification performances of RF, SVM, and CNN models

Algorithm	Kernel function type	Sensitivity	Specificity	Accuracy
RF		92.40%	96.73%	94.47%
SVM	RBF	94.90%	98.74%	96.76%
	Linear	94.38%	98.60%	96.60%
	Polynomial	94.53%	98.56%	96.76%
	Sigmoid	0.73%	99.77%	45.99%
CNN		95.65%	99.51%	97.58%

## Discussion

The application of machine learning-assisted Raman spectroscopy extends beyond breast cancer, showcasing its versatility across different cancer types. Our group has performed this approach on pancreatic cancer and laryngeal cancer [35, 36], which have been successfully verified in both mice and humans. These applications have further validated the technique’s efficacy, demonstrating high accuracy and sensitivity in detecting late-stage cancers, including murine breast cancer models. Despite these promising results in many other cancers, the application of machine learning-assisted Raman spectroscopy for the diagnosis of breast cancer has not yet been explored in late-stage human subjects. Our study is the first work of late-stage breast cancer in the mouse model, which could be a prior exploration of the human model before the clinical trials.

The range of 600–1800 cm^-1^ is notably responsive to molecular alterations, offering insights into the intricate molecular interactions among various bonds [45]. Such spectral analysis is instrumental in identifying changes in biochemical components across different tissues, facilitating the differentiation between normal and pathological states [45]. In Fig. 3, the intensity of the Raman spectrum of health tissue is significantly different than that of cancerous tissue: at the beginning of the Raman shift (600–1200 cm^-1^), the normal has a lower intensity than tumor; from 1200 to 1500 cm^-1^, the tumor has higher intensity, especially the peak of 1442 cm^-1^; after that, the tumor has slightly higher intensity again. The difference in spectral intensity between healthy and cancerous tissues is largely due to the change in lipid content and proteins. Lipid, the major composition in the mammary tissue, has a big Raman cross-Sect. [46]. The Raman bands of lipids at 1302, 1442 cm^-1^ weaken cancerous tissues, which suggests a depletion of lipid reserves during the cancer transformation process. The Raman band of 890 cm^-1^ reflects the structural protein modes of tumors [47]. Compared with healthy tissues, the bands at 936, 1176, and 1573 cm^-1^ are visible in cancerous tissues. These observations imply a dominant protein contribution, underscoring the molecular changes that occur as tissue becomes cancerous.

Several literature sources identify specific molecular structures with specific Raman peaks. The relevant peak assignments for our data are noted in Table 2. The Raman spectrum of normal mammary tissue (Fig. 3a) is dominated by contributions from lipids. The peaks at 1302 and 1442 cm^-1^ reflect the lipid-rich composition of the tissue. The Raman spectra of mammary gland tumors (Fig. 3b) reflect increased protein and reduced lipid compared to normal mammary gland tissue. This shift is evidenced by the presence of more pronounced protein peaks at frequencies of 643, 890, 936, 1035, 1104, 1176, 1355, and 1573 cm^-1^. The variation in peak intensities between normal and cancerous tissues underscores the molecular changes accompanying the transition to a cancerous state, with a marked reduction in lipid content and a concomitant increase in proteins.

**Table 2:** Peak assignments of chemical components and bonds

Peak (cm^− 1^)	Tentative assignment	References
643	C-C twisting (tyrosine)	[48–50]
719	N^+^(CH3)3, choline group, stretch band, C-N (membrane phospholipid head)/nucleotide peak, characteristic for Phosphatidylcholine, sphingomyelin	[51]
740	Adenine/nucleic acid	[52]
847	C-O-C skeletal mode, Monosaccharides	[53]
869	Proline	[50, 54]
890	Structure protein modes of tumors	[55]
936	C-C protein backbone α-helix(proline/glycogen), collagen	[30, 56]
968	Lipids, δ(= CH) wagging	[30, 50]
1035	Collagen	[50, 57]
1066	Proline (collagen)	[23]
1104	Phenylalanine(proteins)	[50, 58]
1117	C-C stretch	[50, 54]
1176	C-H bending tyrosine(proteins)	[55, 56]
1264	δ(= CH) of triglycerides	[30, 50, 59]
1302	δ(CH2) twisting, wagging, phospholipids(lipid)	[30, 54, 56]
1355	Guanine	[47, 60]
1442	Triglycerides	[3, 50]
1573	Guanine, adenine, TRP (protein)	[50, 55]
1645	α-helix (Amide I)	[50]
1656	α-helix (Amide I)	[30, 50]
1720	Amide I	[61]
1738	Lipids	[50]

While these results are promising, they may misconstrue the actual diagnostic ability of Raman spectroscopy. The operating environment in which the spectra were collected, with minimal variability between samples and under the guidance of experienced pathologists, may not fully represent the complexities and challenges encountered in-vivo studies. The introduction of greater variability in sample collection methods and the potential lack of detailed pre-measurement information about the sample in less controlled settings could diminish the diagnostic accuracy. In addition, machine learning algorithms increase the probability of identifying pathological changes owing to their excellent data analysis properties. This study systemically compared three algorithms (RF, SVM, and CNN) with the Raman spectra of mouse breast cancer, which was the first effort in the field of spontaneous-Raman-scattering-aided breast cancer diagnosis [4, 44, 45, 54, 62, 63]; the CNN model typically outperformed the RF and SVM models in the cancerous and non-cancerous tissue classification. Multiple algorithms help validate the results.

Before Raman can be translated for clinical use, many barriers must be overcome. (i) A human tissue Raman data should be collected. (ii) Bio-clean equipment should be designed. To meet this challenge, human samples should be collected under the supervision of trained pathologists and fiber should be sterilizable. Ideally, accompanying algorithms must preprocess and classify the data in near real-time to give an immediate diagnosis in the operating room. In addition, we will also build a benign tumor label since this work only focuses on the normal tissue and malignant tumor.

Our study underscores the distinct Raman spectral features of normal and cancerous tissues and their utility in machine-learning models for diagnosing late-stage breast cancer. These findings pave the way for further research and development to overcome the challenges of translating Raman spectroscopy from a highly controlled research tool to a practical, real-time diagnostic instrument in clinical settings. Emphasizing these aspects can provide a balanced view of the technology’s current achievements and the steps needed to realize its full potential in improving late-stage breast cancer diagnosis and treatment outcomes. The state-of-the-art cancer identification approach is the post-surgery histopathology analysis. In this work, we also did the histopathological test after the resection (Fig. 1c and d). However, there are some disadvantages of the intraoperative pathology analysis, such as high cost and long waiting time (compared to the Raman system). We’ll also try to apply other traditional methods (e.g., MRI) when we do clinical trials in the future.

## Conclusion

This study represented the first effort to systematically compare the effectiveness of three machine learning algorithms (RF, SVM, and CNN) in classifying late-stage animal breast cancer and normal mammary tissue based on their spontaneous Raman scattering signals. The integration of Raman spectroscopy with machine learning techniques enables the automation of tissue classification processes. In particular, the proposed CNN demonstrated the best performance among machine learning approaches used in this study, with an average accuracy, specificity, and sensitivity of 97.58%, 99.51%, and 95.65%, respectively, which is significantly higher performance than previous studies in the field of breast cancer. The differentiation between normal and cancerous mammary tissues was primarily attributed to variations in lipid and protein concentrations, which are critical in the machine learning-based classification of mammary tissues. This underscores the pivotal role of molecular composition, particularly lipids and proteins, in distinguishing between healthy and pathological tissue states through Raman spectroscopy. Overall, the machine learning-assisted Raman spectroscopy demonstrated remarkable accuracy, sensitivity, and specificity in identifying late-stage breast cancerous tissues from non-cancerous tissues, which has the potential to be applied in human diagnosis in the future.

Through the identification of characteristic Raman peaks associated with advanced breast cancer, our approach has successfully demonstrated the potential of this hybrid technology in the accurate diagnosis of this disease stage. This innovation represents a significant leap forward, introducing a novel, efficient method for investigating late-stage breast cancer, which could revolutionize diagnostic practices and potentially improve patient outcomes by facilitating painless and more accurate detection.

## Data Availability

No datasets were generated or analysed during the current study.
